# Molecular Links between Sensory Nerves, Inflammation, and Pain 2.0

**DOI:** 10.3390/ijms241512243

**Published:** 2023-07-31

**Authors:** Éva Szőke, Zsuzsanna Helyes

**Affiliations:** 1 Department of Pharmacology and Pharmacotherapy, Medical School, Eötvös Loránd Research Network, University of Pécs, Szigeti Str. 12, H-7624 Pécs, Hungary; eva.szoke@aok.pte.hu; 2 National Laboratory for Drug Research and Development, H-1117 Budapest, Hungary

Capsaicin-sensitive peptidergic sensory nerves mediate triple actions: besides transmitting sensory and pain signals to the central nervous system (afferent function), they also have local and systemic efferent functions. Pro- and anti-inflammatory neuropeptides (tachykinins, calcitonin gene-related peptide (CGRP), pituitary adenylate cyclase-activating polypeptide (PACAP), somatostatin), and other mediators like purines are released from the activated nerve endings. Their functions on vessels and immune cells determine the role of these nerves in different pathophysiological conditions involved in several diseases, representing unmet medical needs. Furthermore, inflammatory cell-derived mediators can activate or inhibit these nerves. These complex sensory–vascular–immune interactions play important regulatory roles in a range of inflammatory and pain conditions, such as gastrointestinal diseases, endometritis, respiratory diseases, arthritis/osteoarthritis, encephalomyelitis, neuropathic pain, and migraine. Therefore, the present Special Issue compiles papers that explore these molecular mechanisms. In addition, identifying key targets can open promising novel anti-inflammatory and analgesic dug developmental perspectives. The papers published here represent complementary aspects from clinical investigations (original research, systematic reviews, and meta-analyses) to different animal and cellular experimental models.

Miettinen and co-workers showed that chronic pain phenotypes are characterized by specific metabolomic changes, and associated sleep disorders or obesity may influence the metabolome pattern. The most important metabolomic markers and pathways to assign patients to clinical pain phenotypes were determined via a machine learning-based approach and hypothesis-driven analysis, which is likely to promote personalized analgesic therapy [1]. The systematic review of Ren and co-workers summarizes the extensive recent human and animal data regarding lysophosphatidylcholine (LPC) as a potential biomarker and target for chronic pain. They conclude that nerve injury induces oxidative stress, lipid peroxidation, and LPC in the sensory neuronal membrane, leading to hyperexcitability [2]. Another systematic review and meta-analysis of 24,135 rheumatoid arthritis patients demonstrated that Janus kinase (JAK) inhibitors have superiority when improving disease activity and patient-reported outcomes, including pain, as compared to conventional disease-modifying biological therapy [3]. Similar to the joints, the urinary bladder also sees dense peptidergic sensory innervation, which substantially modulates inflammatory and pain mechanisms. The unsatisfactory outcomes of the present diagnoses and treatments of interstitial cystitis and bladder pain patients were identified in the systematic review of Li and colleagues [4]. 

Besides these human results, several papers demonstrated the data obtained in a broad range of experimental systems to obtain deeper insight into pain-related molecular mechanisms. A novel model for the investigation of the transient receptor potential vanilloid 1 (TRPV1) capsaicin receptor pharmacology using a liposomal phospholipid membrane and neuromimetic membrane preparations was described by [5]. Chen and co-workers described interesting interactions between the TRPV1 ion channel on sensory neurons and the P2X7 purinoreceptor on satellite glial cells in a diabetic neuropathic pain rat model [6]. The role of glia-derived cytokine production in neuroinflammation and the related pain was indicated by the antihyperalgesic effect of cocaine- and amphetamine-regulated transcript (CART(55-102)) peptides via dipeptidyl-peptidase 4 inhibition in astrocytes in a carrageenan-evoked rat model of inflammatory pain [7]. Further evidence was provided for the role of neuro-immune interactions and the inflammatory cytokine IL-1 in chronic stress-induced nocifensive behaviors and neuroinflammation in female mice. These results indicated the analgesic potentials of IL-1-blocking drugs in stress-related pain syndromes, such as fibromyalgia, which mainly affects women [8]. Dendritic cell activation was shown to be crucial in both the development and resolution of neuroinflammation via different cell types of the immune system (T and B lymphocytes, macrophages, mast cells) and the resident immune cells of the central nervous system. The neuroprotective effect of dendritic cells with special focus on the function of sirtuins was described the suggesting therapeutic potential of sirtuin-related pathway modification in some neurological diseases [9]. The drug repositioning potential of the centrally acting muscle relaxant tolperisone was suggested by its analgesic activity in neuropathic pain [10].

Proinflammatory neuropeptides, including CGRP, are released from the activated sensory nerve endings, leading to the vasodilation, plasma protein extravasation, oedema formation, and leukocyte accumulation involved in both acute and chronic inflammatory processes. The vasodilator activity of the CGRP-potentiating substance P- and tumor necrosis factor-α alpha-induced oedema and neutrophil accumulation was characterized in the skin microvasculature of mice [11]. Similar to CGRP, PACAP is also pro-inflammatory on the vascular components by inducing strong vasodilation but anti-inflammatory on the cellular components, which makes its overall role in migraine unclear. Several PACAP-induced intracellular pathways and signaling mechanisms were identified in cultured rat trigeminal ganglia primary sensory neurons related to cytokines, oxidative stress, and mitochondrial dysfunction, which are independent of the known and cloned PAC1 receptors [12]. Jana and co-workers described the role of PACAP in the inflamed pig uterus and showed that severe acute endometritis decreased PAC1 receptor expression in the myometrium, leading to decreased contractility [13]. 

Besides neuropeptides, brain-derived neurotrophic factor (BDNF) is also important in neuroinflammation and sensory-vascular-immune crosstalk. Its distribution in the zebrafish sensory organs was described by Aragona et al. (2022 [14]). Since the zebrafish BDNF amino acid sequence is 91% identical to the human peptide, further studies in this model animal could help to clarify the roles of neurotrophins in the sensory system and identify their therapeutic potentials.

Altogether, the *International Journal of Molecular Sciences* team and the two guest editors collected 10 original and 4 review papers, which have been included in this Special Issue. The 14 papers cover a wide range of the molecular mechanisms of the complex sensory–immune–vascular interactions during the inflammatory processes of the central and peripheral nervous systems.
